# Supplementary material for: Caspase-4 promotes metastasis and interferon-γ-induced pyroptosis in lung adenocarcinoma
Source: Commun Biol. 2024 Jun 7;7:699. doi: 10.1038/s42003-024-06402-3 (PMC11161495; doi:10.1038/s42003-024-06402-3)
Supplement: Supplementary file 2 — Supplementary Information [file 42003_2024_6402_MOESM2_ESM.pdf]

Table S1. *CASP4* mRNA expression and clinicopathological factors in the lung adenocarcinoma patients.

	high *CASP4* mRNA (n=29)	low *CASP4* mRNA (n=27)	P-value
Age (mean ±SD)	67.24(±12.17)	69.22(±7.82)	0.48 (a)
Gender			0.28 (b)
Male	19	13	
Female	10	14	
Stage			0.004 (b)
I-II	21	27	
III-IV	8	0	
Historical Grade			0.46 (b)
I	2	3	
II	15	17	
III	12	7	
Ⅳ	0	0	
Pathological Tumor Size	29.10 (±23.13)	25.85 (±12.10)	0.52 (a)
CASP4 mRNA (FPKM)	100.3(±40.66)	50.12(±11.37)	< 0.001 (a)
CEA (ng/mL)	6.58 (±8.53)	3.13 (±2.08)	0.04 (a)
CRP (ng/mL)	0.38 (±0.82)	0.17 (±0.30)	0.21 (a)
WBC (/µL)	6277 (±1361)	5286 (±1636)	0.27 (a)
Neutrophil (/µL)	3833 (±1146)	3768 (±1289)	0.84 (a)
Lymphocyte (/µL)	1735 (±600.2)	1441 (±459.6)	0.04 (a)
Plt (/µL)	24.5 (±5.52)	22.5 (±4.23)	0.14 (a)
Alb (g/dL)	4.29 (±0.42)	4.27 (±0.29)	0.90 (a)

(a) Student's t-tests

(b) Chi-squared test

Table S2 (Chiba et al)

Primers for quantitative real time PCR	sequence
hGAPDH FWD	GTCTCCTCTGACTTCAACAGCG
hGAPDH REV	ACCACCCTGTTGCTGTAGCCAA
hCASP4 FWD	AAGAGAAGCAACGTATGGCAGGAC
hCASP4 REV	GGACAAAGCTTGAGGGCATCTGTA
hSERPINE1 FWD	CCTCCAGCAGCTGAATTCCT
hSERPINE1 REV	GGGTTTCTCCTCCTGAAGTTCT
hPTGS2 FWD	CGGTGAAACTCTGGCTAGACAG
hPTGS2 REV	GCAAACCGTAGATGCTCAGGGA
hEPHA2 FWD	ACTGCCAGTGTCAGCATCAACC
hEPHA2 REV	GTGACCTCGTACTTCCACACTC
mActb FWD	CATCCGTAAAGACCTCTATGCC
mActb REV	ATGGAGCCACCGATCCACA
mCasp11 FWD	TTTTACTCTGTCAAGCTGTCTT
mCasp11 REV	AGGGTGTTTGTTTTCAGCCAT
mEpha2 FWD	GGCTGTACTCAAGTTTACCACCG
mEpha2 REV	CCGCTTTCAGTGTCTTGATGGC
mPtgs2 FWD	GCGACATACTCAAGCAGGAGCA
mPtgs2 REV	AGTGGTAACCGCTCAGGTGTTG
mCd31(Pecam1) FWD	CCAAAGCCAGTAGCATCATGGTC
mCd31(Pecam1) REV	GGATGGTGAAGTTGGCTACAGG
mCd34 FWD	AGGACAGCAGTAAGACCACACC
mCd34 REV	GTGTGGAGTTCCAGAGCCTGAA

Primers for for knockdown with shRNA	sequence
shCASP4 FWD	AAGCTTTGTCCTCATGAAGAATT
shCASP4 REV	AATTCTTCATGAGGACAAAGC
shSTAT1 FWD	GAGATGTGAATGAGAGAAATACA
shSTAT1 REV	TGTATTTCTCTCATTCACATC

List of antibodies

Antibody	Isotype	Suppliers	Usage
anti CASP4 (M029-3)	mouse	MBL	WB/fluorescent immunostaining
anti β-actin (#643807)	rabbit	BioLegend	WB
anti p65 (#8242)	rabbit	Cell Signalling Technology	WB/fluorescent immunostaining
anti p-p65 (Ser536) (#3033)	rabbit	Cell Signalling Technology	WB
anti p-STAT1 (Tyr701) (#9167)	rabbit	Cell Signalling Technology	WB
anti STAT1 (#9172)	rabbit	Cell Signalling Technology	WB
anti CD31 (#77699)	rabbit	Cell Signalling Technology	Immunohistochemistry (Paraffin)
anti Gasdermin D (#97558)	rabbit	Cell Signalling Technology	WB
anti EPHA2 (sc-398832)	mouse	Santa Cruz Biotechnology	WB
anti COX2/PTGS2 (#12282)	rabbit	Cell Signalling Technology	WB
anti rabbit IgG, HRP-linked (#7074)	goat	Cell Signalling Technology	WB
anti mouse IgG, HRP-linked (#7076)	horse	Cell Signalling Technology	WB
Alexa Fluor 488 anti-rabbit IgG (#406416)	donkey	Biolegend	fluorescent immunostaining
Alexa Fluor 647 anti-rabbit IgG (#406414)	donkey	Biolegend	fluorescent immunostaining

List of reagents

Reagents	Suppliers
LPS (L9143)	Sigma-Aldrich
human IFN-γ (099-06113)	Wako
human TNF-α (201-15264)	Wako
Ferrostatin-1 (S7243)	Selleckchem
C11 BODIPY 581/591 lipid ROS probe	Dojindo

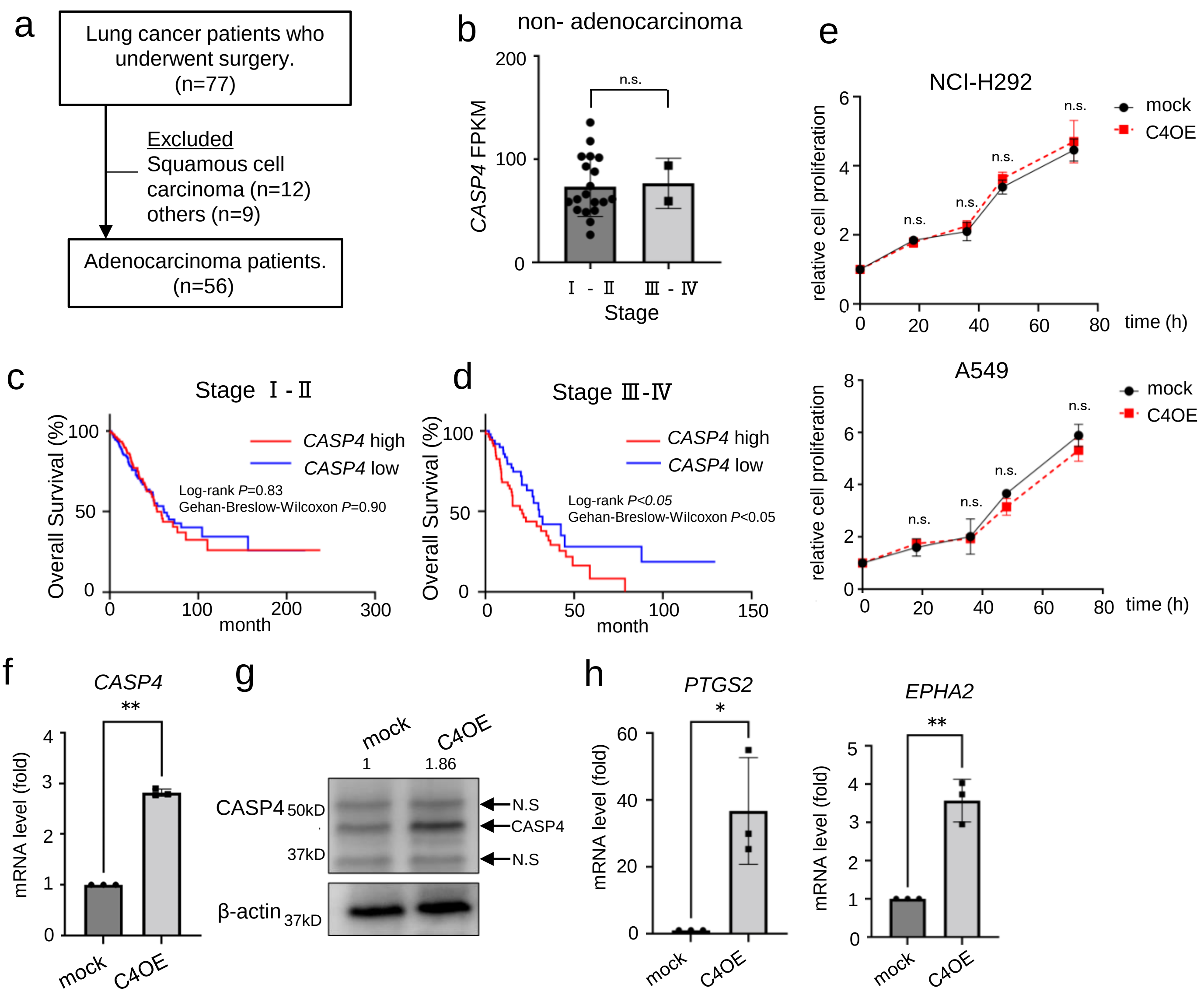

Figure S1. *CASP4* overexpression upregulates cell migration, angiogenesis, and related genes (Related to Figure 1).

(a) Lung adenocarcinoma patient who underwent surgical resection at Aichi Cancer Center Hospital selection flowchart. (b) Comparison of *CASP4* mRNA expression at each stage (stages Ⅰ–Ⅱ vs. Ⅲ–Ⅳ) based on RNA sequencing (RNA-seq) data of non-adenocarcinoma tissues (n = 21). (c) and (d) Comparison of overall survival by *CASP4* mRNA expression based on The Cancer Genome Atlas Program (TCGA) and Lung Adenocarcinoma (LUAD) database analysis (stage Ⅰ–Ⅱ: n = 399, stage Ⅲ–Ⅳ: n = 110); *CASP4* high is defined as mRNA expression z-scores relative to all samples (log RNA-Seq V2 RSEM) of >0.08, and *CASP4* low is defined below that. (e) Cell proliferation of *CASP4* overexpressing (C4OE) cells and control cells with normal *CASP4* expression cells (mock). (f) Comparison of *CASP4* mRNA expression between mock and *CASP4* overexpressing (C4OE) cells by real-time quantitative PCR (RT-qPCR). (g) Representative images of the immunoblotting analysis of CASP4 protein levels in mock and C4OE cells. (h) Representative angiogenesis and cell motility-related gene upregulated by C4OE by RT-qPCR. *P < 0.05, **P < 0.01 compared with mock. N.S: non-specific, Densitometry values are shown above each blot.

Figure S1

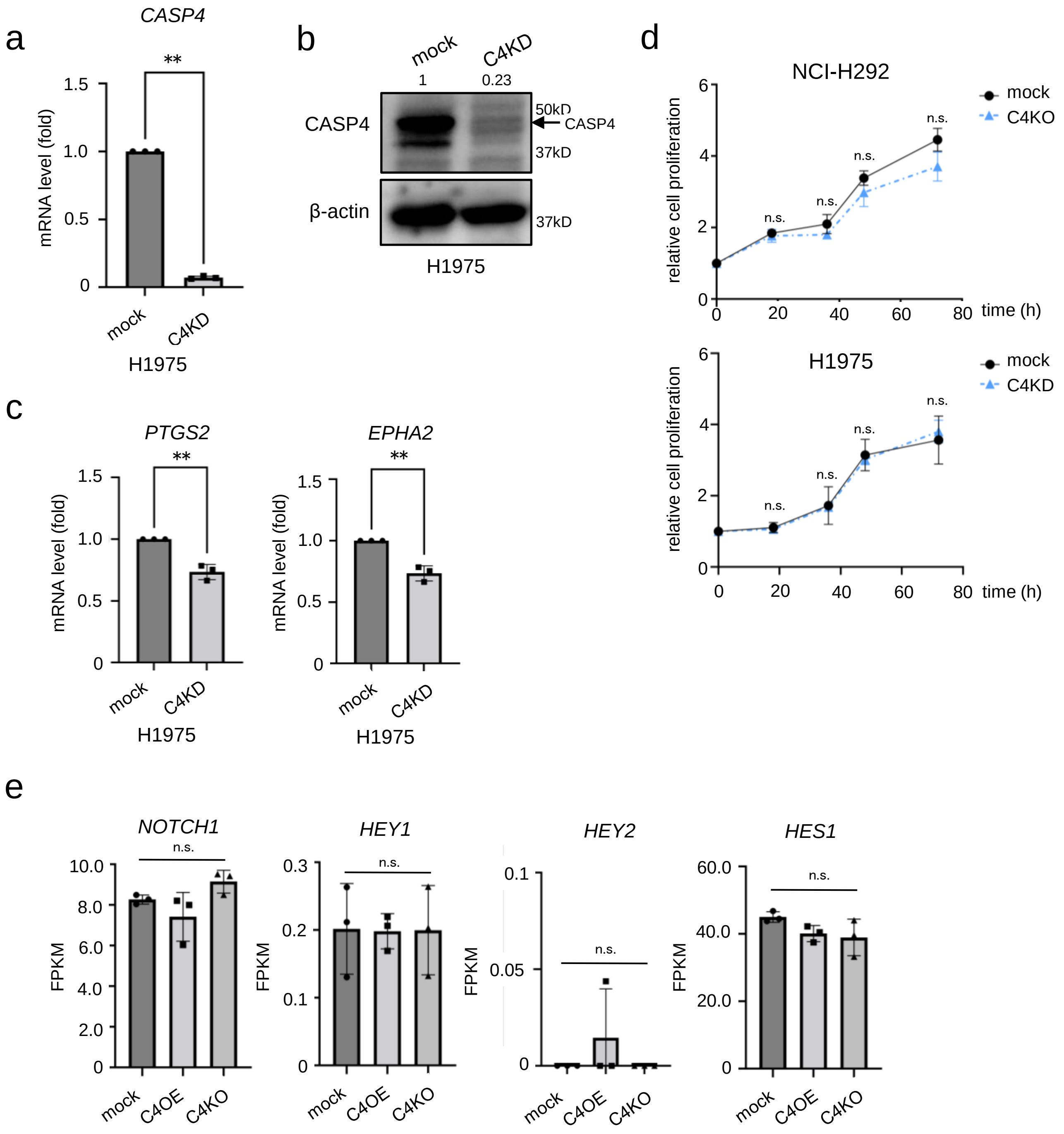

Figure S2. ***CASP4*** **knockdown decreases angiogenesis and cell motility-related genes (Related to Figure 2).**

(a) Comparison of *CASP4* mRNA expression between mock and *CASP4* knockdown (C4KD) cells by RT-qPCR. (b) Representative images of the immunoblotting analysis of CASP4 protein levels in mock and C4KD cells. (c) Representative angiogenesis and cell motility-related gene downregulated by *CASP4* knockdown by RT-qPCR. (d) Cell proliferation of *CASP4* knockout (C4KO) or C4KD cells and control cells with normal *CASP4* expression cells (mock). (e) RNA-seq analysis of *NOTCH1*, *HEY1*, *HEY2*, and *HES1* in mock, CASP4 overexpression (C4OE) and C4KO NCI-H292 cells. Each gene is presented as fragments per kilobase of exon million mapped reads (FPKM). $^*P < 0.05$, $^{**}P < 0.01$ compared with mock. n.s. : not significant. Densitometry values are shown above each blot.

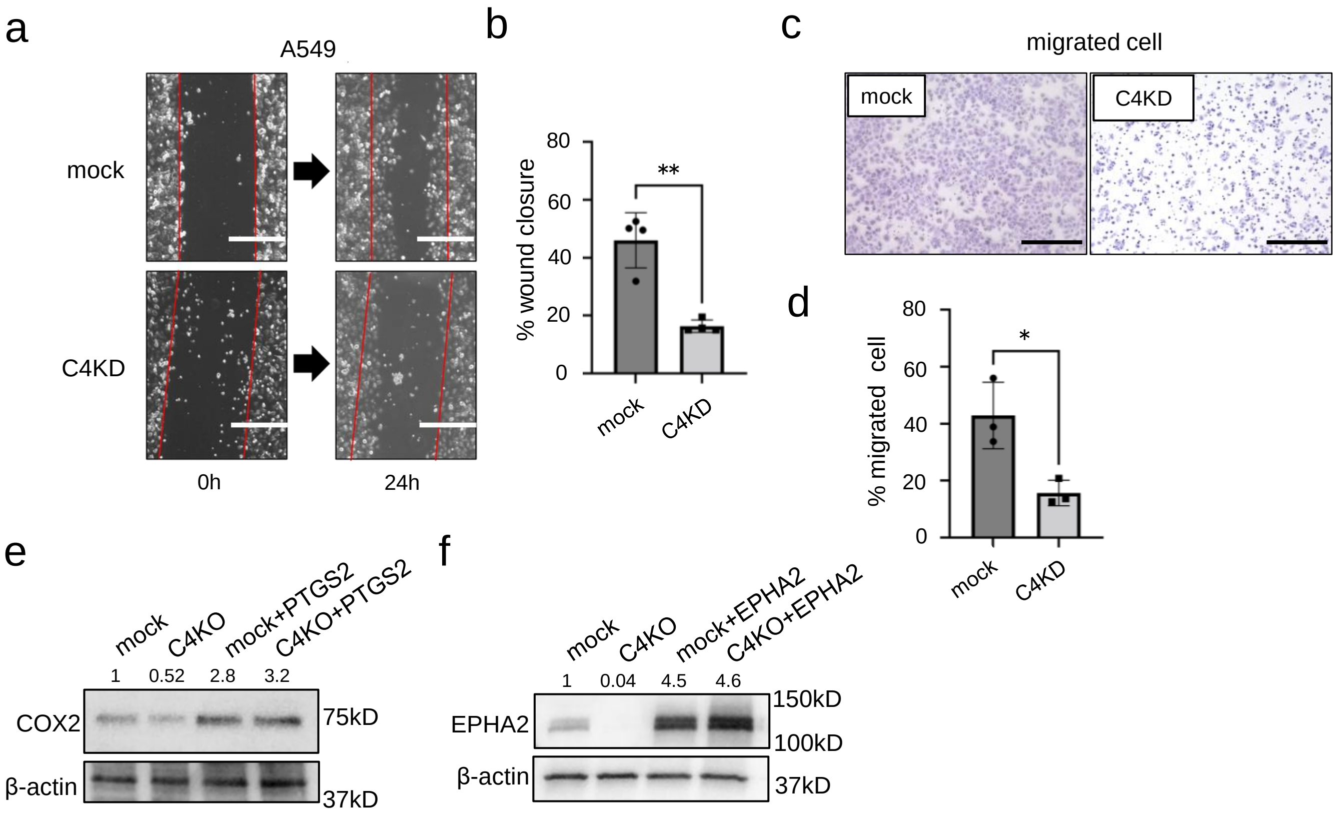

Figure S3. CASP4 promotes cell migration (Related to Figure 3).

(a) Representative images of the wound healing assay of mock or C4KD A549 cells at 0 and 24 h. Scale bar, 500 µm. (b) Quantitative analysis of cell motility in A by mock or C4KD A549 cells as indicated by the rate of % wound closure of a scratched area at 24 h (n = 4). (c) Representative images of cell migration assay of mock or C4KD A549 cells at 48 h. Scale bar, 100 µm. (d) Quantitative analysis of cell migration in E by mock or C4KD A549 cells as indicated by the rate of % migrated cell at 48 h(n = 3). (e) Representative images of immunoblotting analysis of COX2 protein in mock and C4KO, and cells with rescued PTGS2 gene in NCI-H292 cells. (f) Representative images of immunoblotting analysis of EPHA2 protein in mock and C4KO, and cells with rescued EPHA2 gene in NCI-H292 cells. *P < 0.05, **P < 0.01 compared with the mock. Densitometry values are shown above each blot.

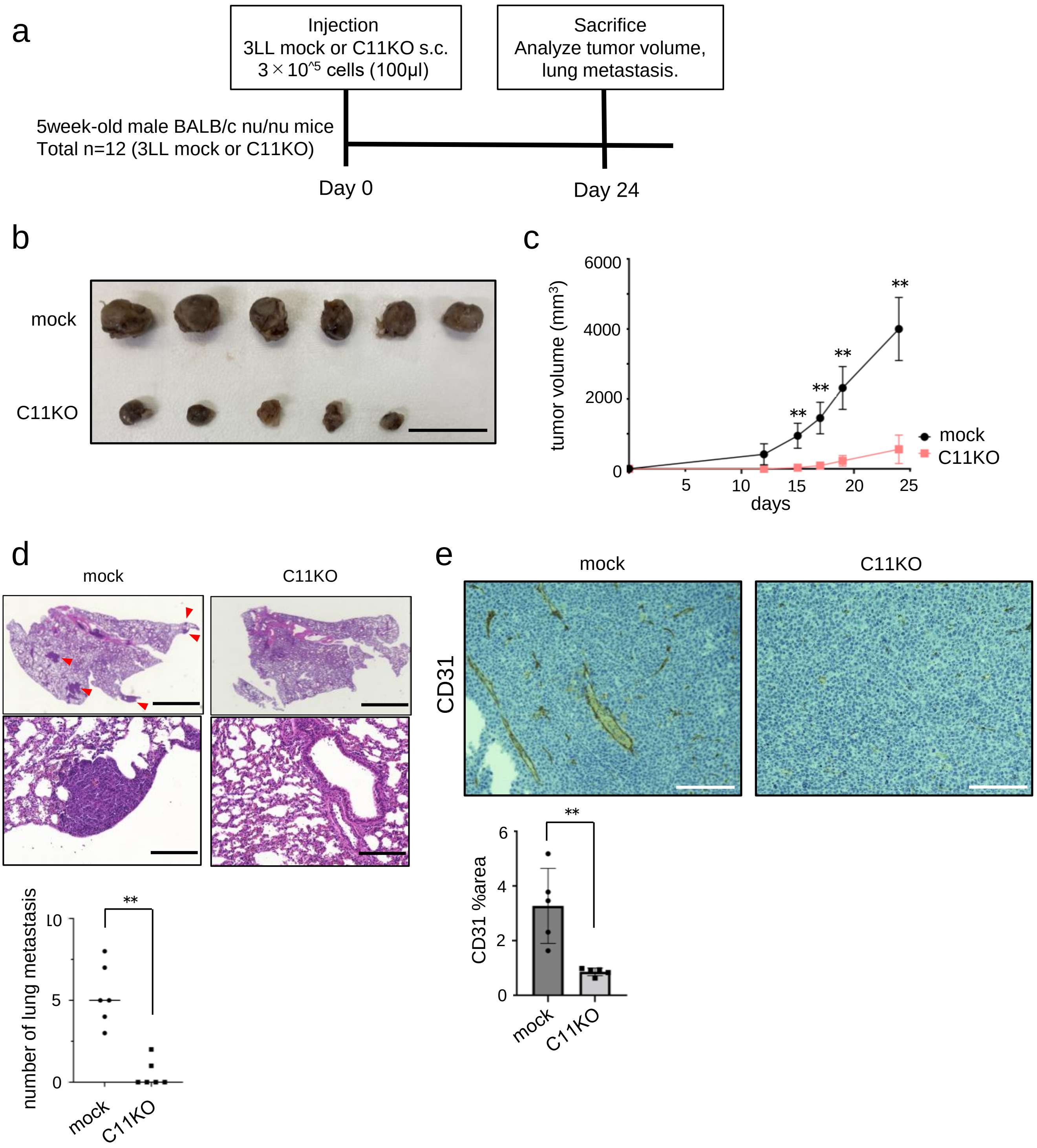

Figure S4. *Casp11* knockout attenuates tumor growth and angiogenesis to inhibit metastasis in immunodeficient mice (BALB/c nu/nu). (Related to Figure 4).

(a) The diagram showing the time course of the allogenic mouse model. (b) Representative macroscopic images of primary tumors of mock or C11KO 3LL cells at 24 days after injection. Scale bar = 30 mm. (c) Primary tumor volume of mock and C11KO 3LL cells injected in the mouse (n = 12). (d) Representative microscopy images of HE staining of the left lung of mock and C11KO 3LL cells (upper panel). Scale bars in upper and lower pictures indicate 2,500 and 250 μm, respectively. The number of tumor metastases in the left lung in mice injected with mock and C11KO cells (lower panel). Red arrow indicates lung metastasis. (e) Representative microscopy images of CD31 immunostaining of the primary tumors of mock and C11KO cells (upper panel). Scale bar, 100 μm. Quantitative analysis of CD31 positive area of primary tumors of mock and C11KO cells (lower panel). *P < 0.05, **P < 0.01 compared with mock.

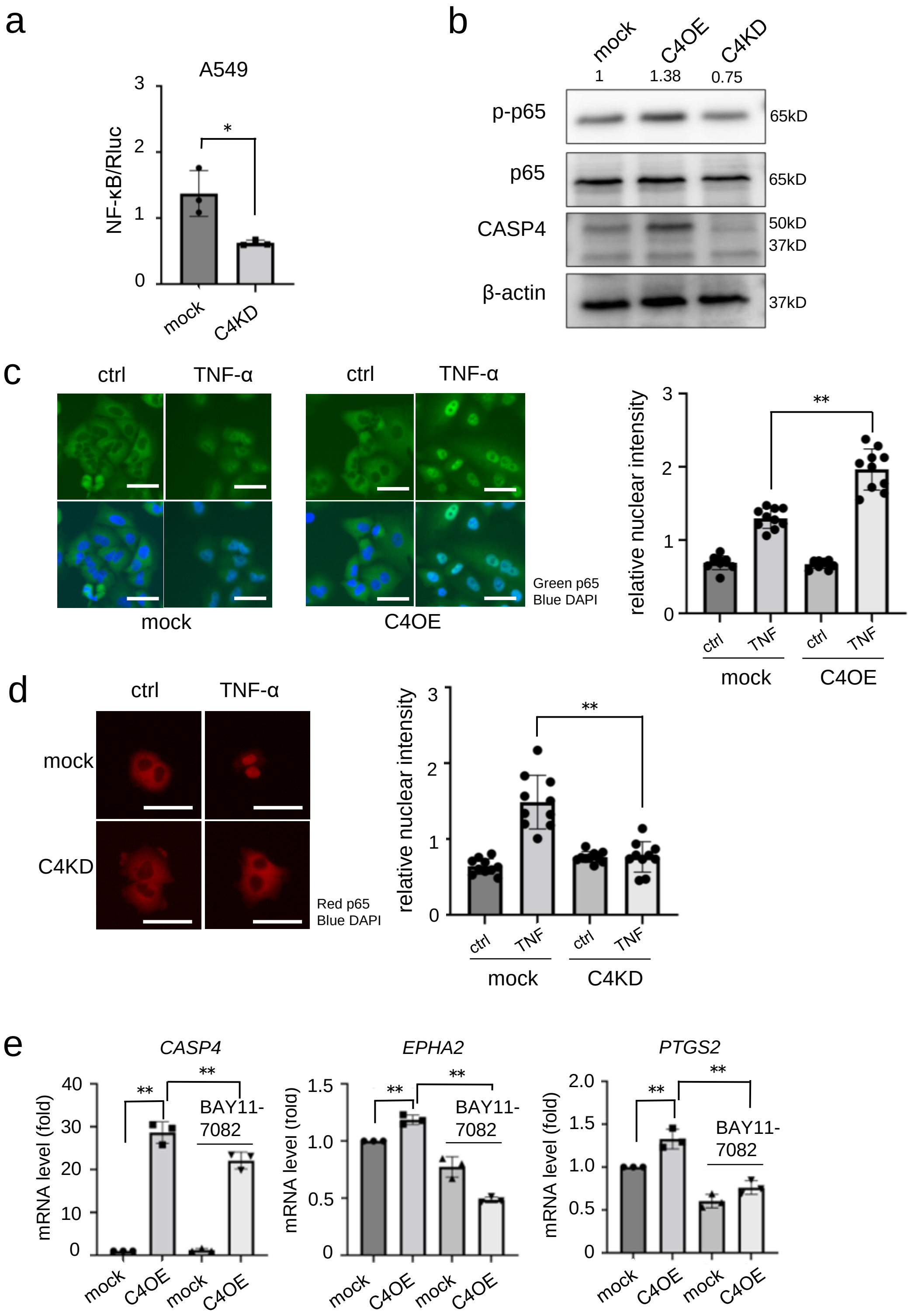

Figure S5. NF-κB pathway contributes to the up-regulation of genes related to angiogenesis and cell migration in A549 cells (Related to Figure 5).

(a) Relative comparison of luciferase activity between mock and C4KD A549 cells transfected with an NF-κB expression plasmid. (b) Immunoblotting analysis of phospho-p65, p65, and CASP4 protein levels in mock and C4KD A549 cells. (c)(d) Expression and localization of p65 by immunofluorescence staining in mock and C4OE or C4KD A549 cells treated with 20 ng/ml of TNF-α for 1 h. Scale bar, 20 μm. Quantification of nuclear/cytoplasmic ratio of p65 intensity (n=10). (e) mRNA levels of CASP4, EPHA2, and PTGS2 in mock, and C4OE with or without the treatment of NF-κB inhibitor BAY11-7082 (10 μM) for 8h in A549 cells by RT-qPCR. *P < 0.05, **P < 0.01 compared with mock. Densitometry values are shown above each blot.

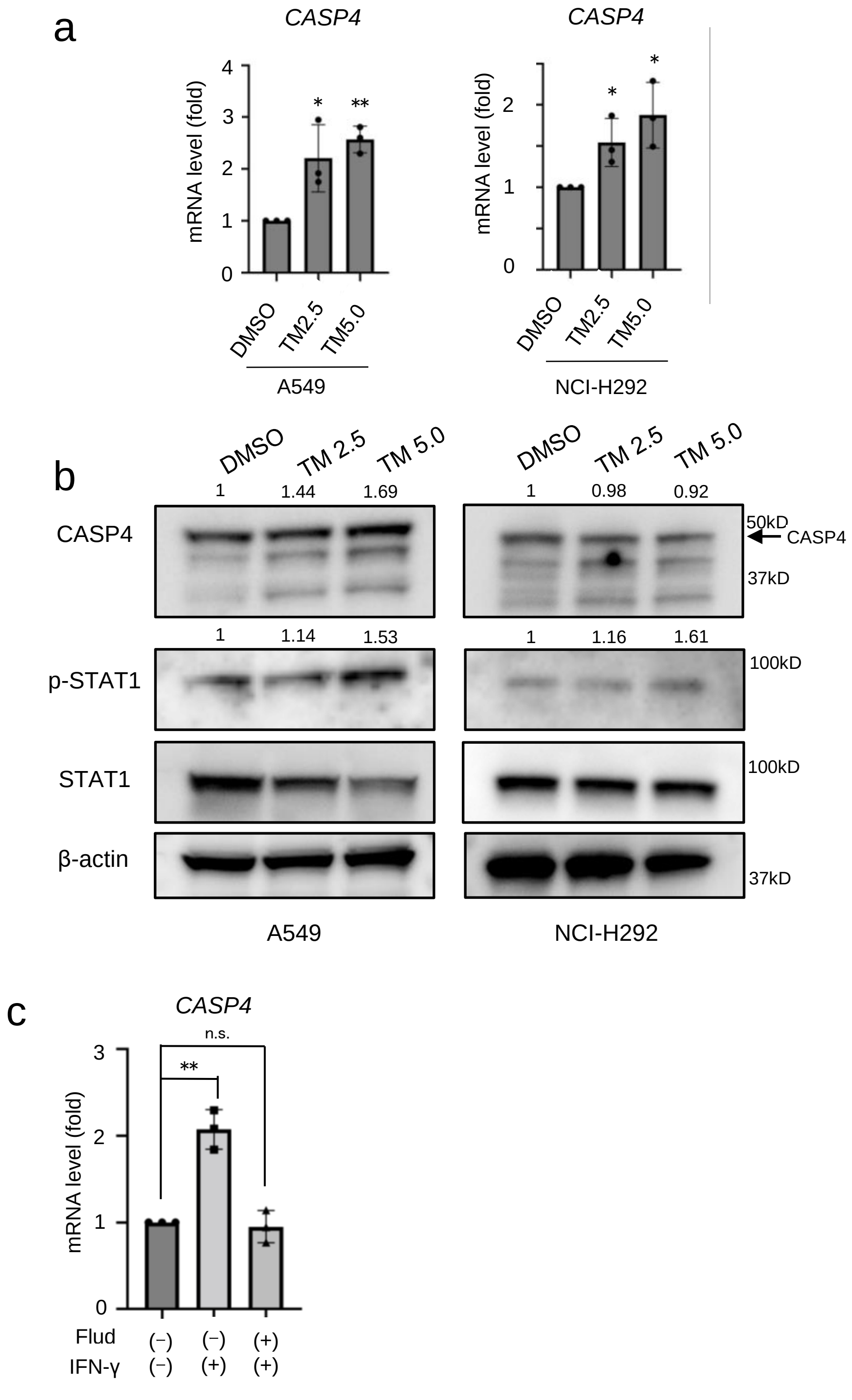

Figure S6. CASP4 is induced by ER stress (Related to Figure 6).

(a) Induction of *CASP4* mRNA by the ER stress inducer tunicamycin (TM, µg/ml) for 12h by RT-qPCR. (b) Immunoblotting analysis of CASP4 and p-STAT1 / STAT1 treated with PBS and TM (µg/ml) for 24h in A549 and NCI-H292 cells. (c) Suppression effect of *CASP4* mRNA by STAT1 inhibitor, fludarabine (Flud). NCI-H292 cells treated with IFN-γ 10 ng/ml for 24h with or without of Flud 5 µM. Fludarabine was pretreated for 3h before IFN-γ treatment. n.s : not significant, *P < 0.05, **P < 0.01 compared with mock.

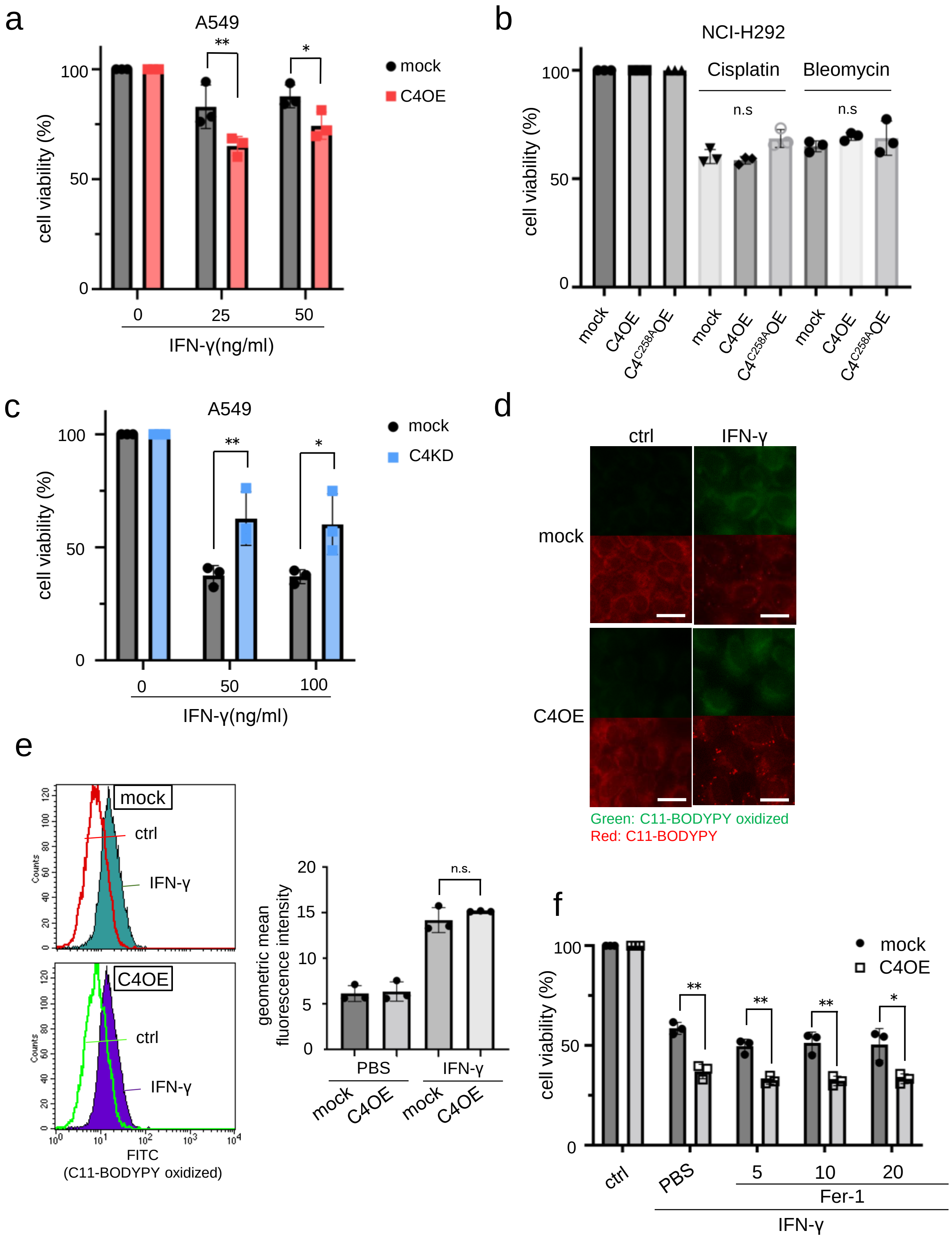

Figure S7. CASP4 expression increases the susceptibility of IFN-γ to induce cell death (Related to Figure 7).

(a) The rate of viable cells of mock, C4OE A549 cells treated with 100 ng/ml of IFN-γ for 72h measured by CCK-8 assay. (b) The rate of viable cells of mock, C4OE, and $C4^{C258A}OE$ NCI-H292 cells by cytotoxic reagents (cisplatin at 10 μM, and bleomycin at 50 μg/ml stimulated for 24 h) using CCK-8 assay. (c) The rate of viable cells of mock, C4KD A549 cells treated with 50 or 100 ng/ml of IFN-γ for 72h measured by CCK-8 assay. (d)(e) Detection of lipid peroxide by green fluorescence of C11-BODYPY in NCI-H292 cells treated with 100 ng/ml of IFN-γ for 72h. Scale bar, 20 μm. (f) Cell viability in NCI-H292 cells stimulated with 100 ng/ml IFN-γ for 72h with or without ferrostatin-1 (Fer-1, μM) using CCK-8 assay. Fer-1 was pretreated for 3h before IFN-γ treatment. n.s.: not significant, $*P < 0.05$, $**P < 0.01$ compared with mock.

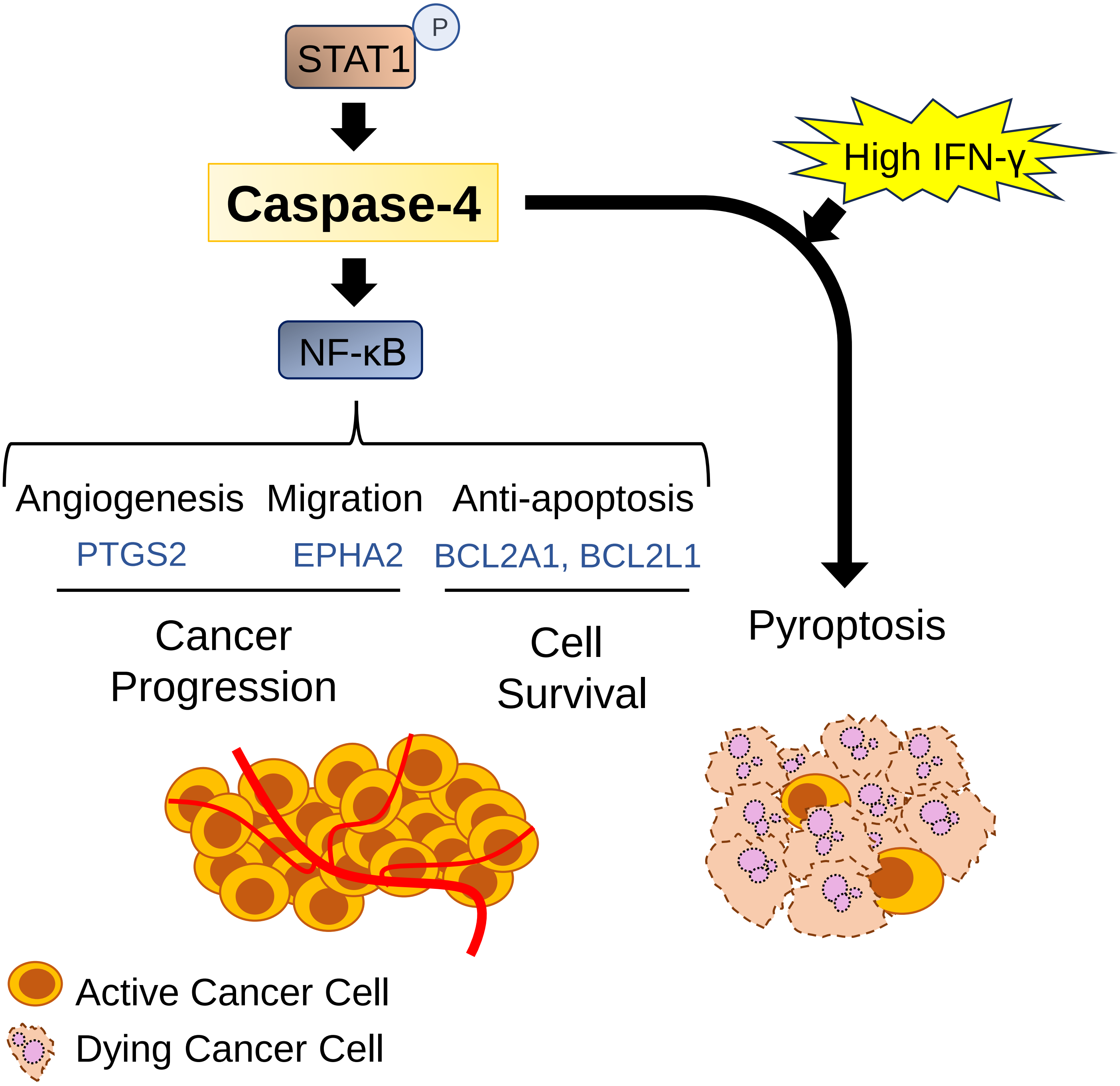

Figure S8. Graphical Abstract

Fig. 7c

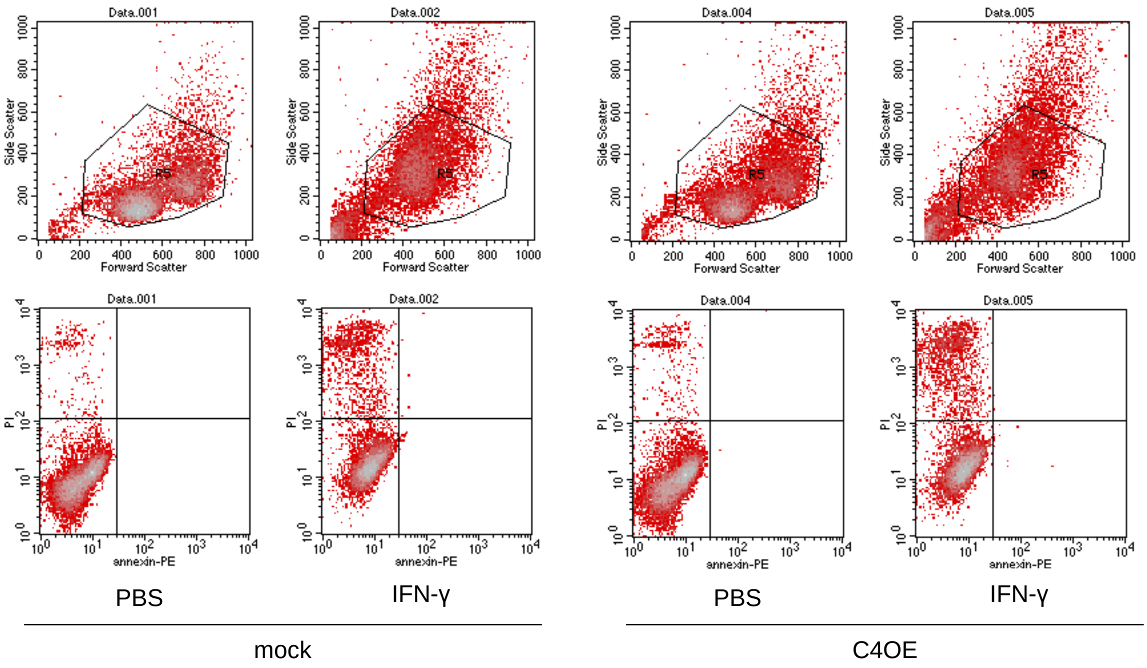

Figure S9. FACS gating strategy corresponding to Figure 7c.

Fig. 1b

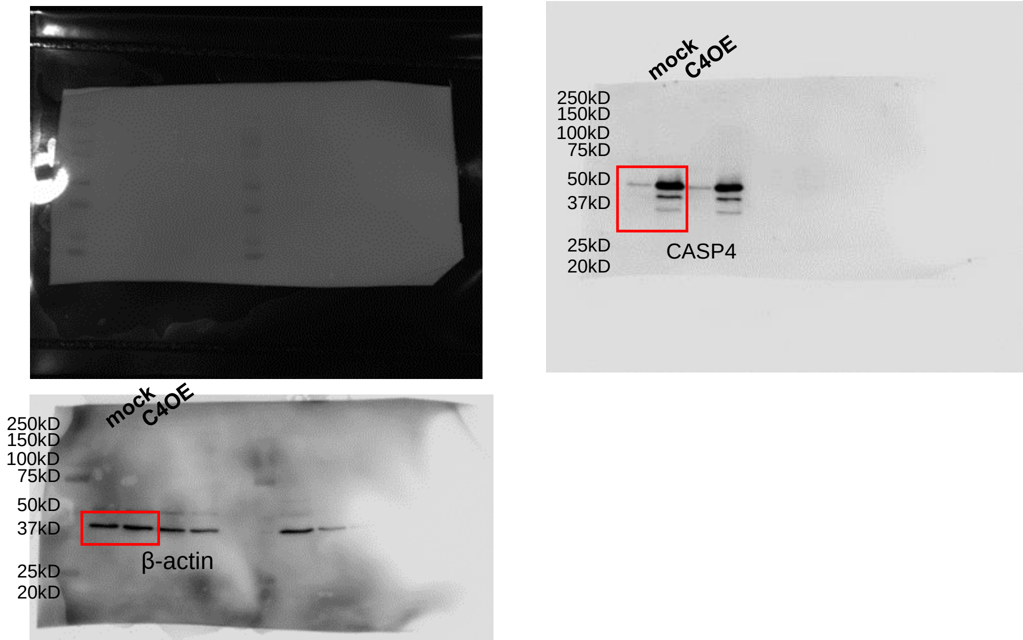

Fig. 2a

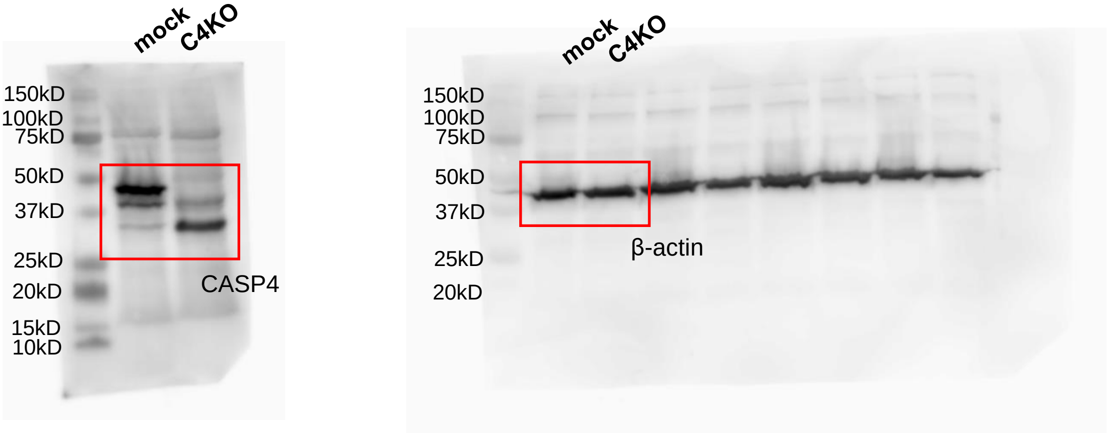

Fig. 4b

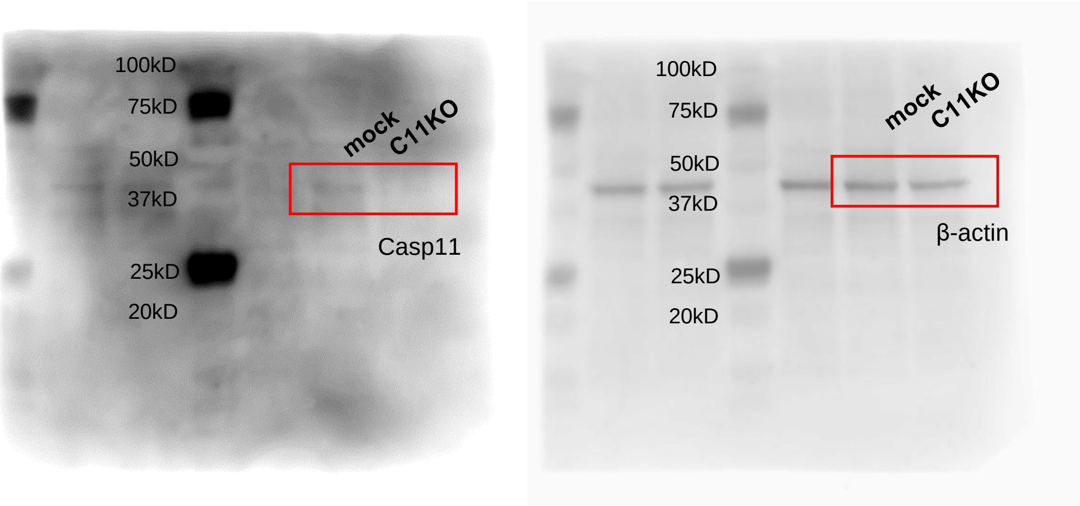

Figure S10. Full Western blot images corresponding to Figure 1 to Figure 4.

Fig. 5d

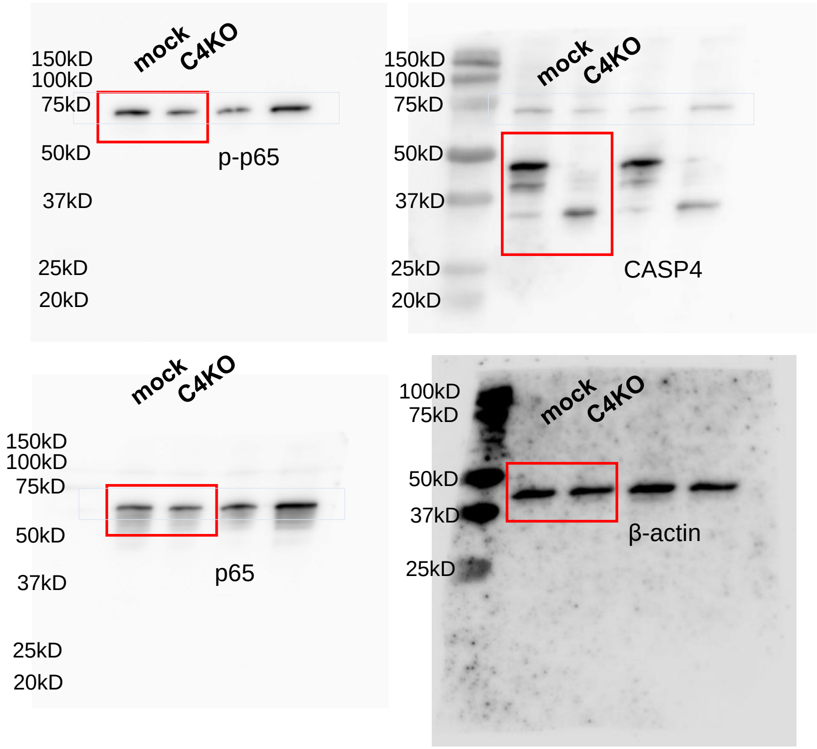

Fig. 5f

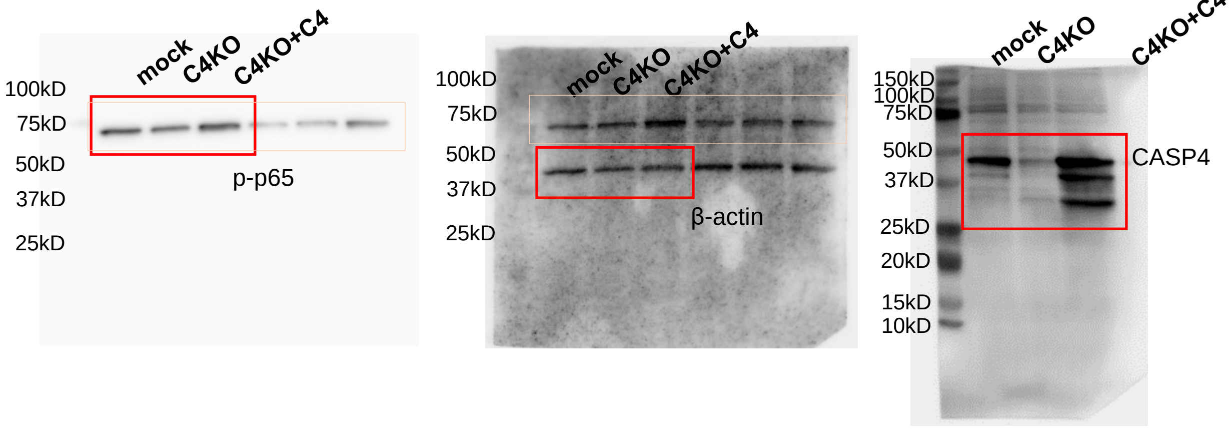

Fig. 5h

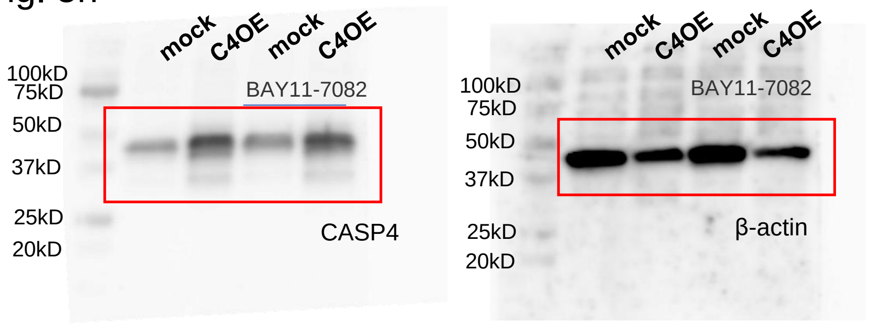

Figure S11. Full Western blot images corresponding to Figure 5

Fig. 6d

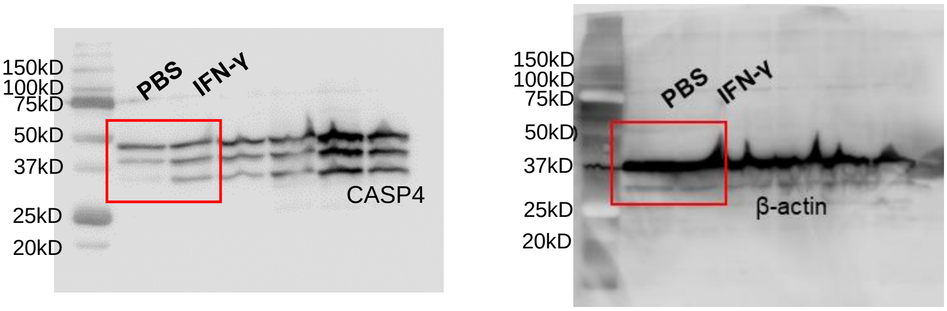

Fig. 6h

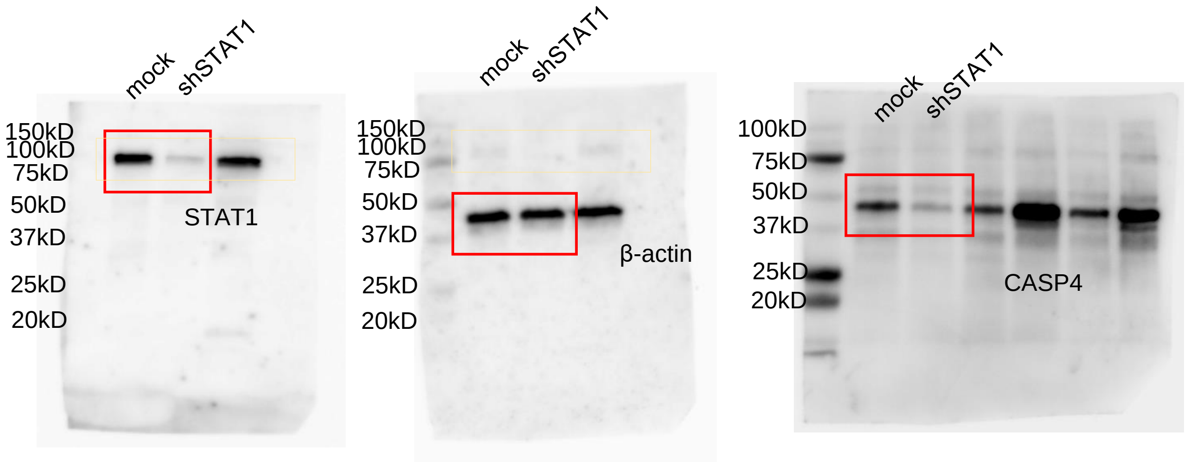

Fig. 7f

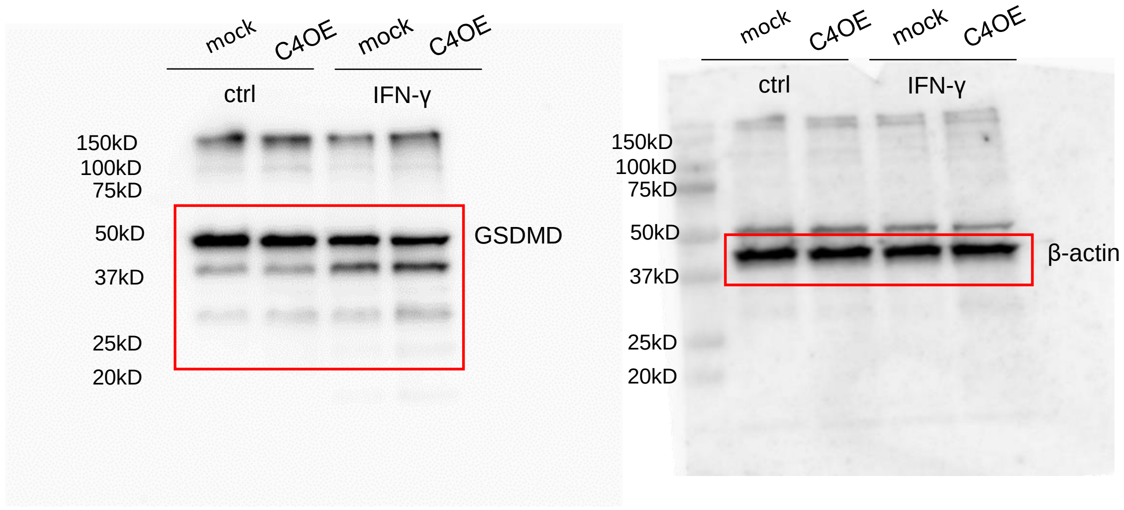

Figure S12. Full Western blot images corresponding to Figure 6 and Figure 7
