# Supplementary material for: Caspase-4 promotes metastasis and interferon-γ-induced pyroptosis in lung adenocarcinoma
Source: Commun Biol. 2024 Jun 7;7:699. doi: 10.1038/s42003-024-06402-3 (PMC11161495; doi:10.1038/s42003-024-06402-3)
Supplement: Supplementary file 3 — Description of Additional Supplementary Files [file 42003_2024_6402_MOESM3_ESM.pdf]

Description of Additional Supplementary Files

File name: Supplementary Data 1-7
Description: The source data behind the graphs in the paper.
